# The reasons why Flemish low-risk nulliparous pregnant women choose a specific antenatal care provider: A descriptive qualitative study

**DOI:** 10.18332/ejm/224241

**Published:** 2026-10-15

**Authors:** Annelore Cloostermans, Ayse Akalin, Joeri Vermeulen, Maaike Fobelets

**Affiliations:** 1 Faculty of Medicine and Pharmacy, Vrije Universiteit Brussel, Brussels, Belgium; 2 Faculty of Medicine and Pharmacy, Biostatistics and Medical Informatics Research Group, Vrije Universiteit Brussels, Brussels, Belgium; 3 Brussels Institute for Teacher Education, Vrije Universiteit Brussel, Brussels, Belgium; 4 Faculty of Health Sciences, Department of Nursing, Düzce University, Düzce, Türkiye; 5 Faculty of Science, Technology and Medicine, Department of Life Sciences and Medicine, University of Luxembourg, Eschsur-Alzette, Luxembourg; 6 Department of Healthcare, Erasmus Brussels University of Applied Sciences and Arts, Brussels, Belgium

**Keywords:** antenatal care, low-risk pregnancy, nulliparous, decision making, healthcare choices

## Abstract

**INTRODUCTION:**

In Belgium, low-risk pregnant women have the option of consulting an obstetrician, midwife, or general practitioner for antenatal care. Although consultations with midwives have increased over the years, obstetricians remain the most frequently consulted providers, even for low-risk pregnancies. However, the reasons behind provider choice remain unclear. The aim of this study was to ascertain the reasons why Flemish low-risk nulliparous pregnant women choose a specific antenatal care provider.

**METHODS:**

A descriptive qualitative study was carried out in Flanders, Belgium, between March and May 2024, with Flemish low-risk nulliparous pregnant women. Semi-structured individual interviews were conducted by a female midwife experienced in qualitative research. An open-ended interview guide was used to explore participants’ decision-making processes, experiences with antenatal care providers, expectations of care, and perceived roles of antenatal care providers. Braun and Clarke’s six-step thematic analysis was applied to analyze the data in NVivo^®^.

**RESULTS:**

Thirteen women were interviewed. Their choice of antenatal care provider was shaped by perceptions of medical expertise, accessibility, social influences, and structural constraints. Obstetricians were commonly preferred for their specialization and access to technology, whereas midwives were valued for relational and emotional support once women became aware of their role. Five overarching themes were identified: trust in professional expertise, practical accessibility of care, influence of social environment and informational gaps, need for relational continuity and emotional support, and structural and organizational constraints.

**CONCLUSIONS:**

The decision-making process for choosing an antenatal care provider is multidimensional and varies among Flemish low-risk nulliparous women. Further research is needed to explore healthcare professionals’ perspectives on low-risk antenatal care pathways and to examine how different models of antenatal care shape women’s decision-making processes and care experiences.

## INTRODUCTION

Antenatal care is essential for ensuring the health and wellbeing of both the pregnant woman and her unborn child^1^. The World Health Organization (WHO) recommends a comprehensive model of antenatal care, emphasizing early and regular check-ups to monitor fetal development, manage potential complications, and provide essential health education^2^. In Belgium, as in many other high-income countries, expectant mothers can choose their primary antenatal care provider (ACP), including obstetricians, midwives, and, to a less extent, general practitioners (GPs)^3^. Most women choose an obstetrician as their primary ACP. Midwives, who mainly operate in primary care and manage primarily low-risk, physiological pregnancies, are consulted by a smaller portion of the population^3,4^. While pregnant women are legally entitled to choose their ACP, key medical procedures – such as the three recommended ultrasounds – must be performed by an obstetrician, the only authorized provider for these exams^3^. The Belgian compulsory health insurance, partially, reimburses specialist care without prior approval, regardless of pregnancy type^5^. This makes specialized care highly accessible, leading most women to consult obstetricians directly for antenatal care.

In Belgium, ten antenatal consultations for nulliparous women and seven for multiparous women with a low-risk pregnancy are recommended, combining visits with obstetricians, midwives, and GPs^6^. However, data from 2016 show a high utilization of antenatal care among low-risk pregnant women, with 85.08% attending more than ten consultations and only 3.67% having fewer than seven. On average, low-risk women had a median of two consultations with a midwife and ten with an obstetrician^7^. Despite the regulatory framework, the choice for a specific ACP is influenced by multiple factors^8,9^. International literature indicates that both the organization of the healthcare system and personal factors influence the choice for a specific ACP. These choices are further influenced by socioeconomic status, demographics, whether healthcare providers actively inform patients about their healthcare rights and entitlements^8,9,10-14^, geographical accessibility, financial accessibility, and the availability of healthcare professionals^13,15,16^.

In addition to organizational factors, several personal factors influence the choice of ACP. These include women’s understanding of the role and legal competence of an ACP^4,11,16^, and the ACP’s perceived expertise and associated safety – where some women feel safer with obstetricians, especially following previous complications or in cases of childbirth-related fear, while others trust midwives or GPs depending on their views on care^8,13,14,16-19^. Personal medical beliefs also play a role: those who see pregnancy as a natural process tend to prefer midwives, while women who anticipate interventions like epidurals or cesarean sections are more likely to choose obstetricians^12,13,16,17,20,21^. Previous experiences with an ACP further shape choices, with many women opting for the same type of professional when they had positive, continuous, and personalized care in the past^10,13,16,19^. Lastly, personal environment and identity also play a role, as choices are often influenced by recommendations from friends or family, or preferences for female providers, such as midwives^10,13,16,20^.

A Belgian report by Benahmed et al.^15^ on integrated antenatal care for low-risk pregnancies highlighted the following elements influencing women’s choice of an ACP. These include established habits, trust and familiarity with the provider, appreciation of certain personality characteristics, finding the provider by coincidence, a sense of compatibility and comfort, availability, geographical proximity, and the link to a hospital of preference. Although existing research has been performed about the choice of an ACP, most studies include mixed-risk pregnancies and multiparous women, whose choices may be influenced by prior experiences^10,11,12,16^. Therefore, this study focuses specifically on low-risk, nulliparous pregnant women in Flanders, aiming to ascertain the reasons behind their choice of an ACP. By identifying key factors influencing their decisions, the research seeks to deepen understanding of maternal preferences within the Flemish maternity care system.

## METHODS

### Study design

This qualitative descriptive study, using semi-structured interviews, was conducted in Flanders, Belgium, between March and May 2024. The Standards for Reporting Qualitative Research (SRQR) checklist was used as a guide for the comprehensive reporting of the qualitative study data^22^ (Supplementary file S1).

### Setting and participants

Participants were recruited through purposive sampling within primary care settings in Flanders, Belgium. To ensure adequate enrolment, additional participants were recruited via snowball sampling and outreach through personal networks. Pregnant women who were interested in participating were invited to contact the research team in response to a recruitment flyer.

Eligibility criteria included being a low-risk pregnant woman who: 1) resided in Flanders; 2) was aged 18–39 years; 3) was up to 27 weeks gestation; 4) was nulliparous; 5) demonstrated sufficient proficiency in speaking, reading, and understanding Dutch; 6) had attended at least one antenatal consultation with a midwife and/or an obstetrician; 7) agreed to schedule an interview within two weeks; and 8) provided informed consent.

Midwives, obstetricians, and GPs were excluded from participation to focus solely on the experiences and perceptions of pregnant women receiving care. The gestational age was restricted to the second trimester (maximum 26 weeks pregnant at time of study notification) in order to capture determinants of ACP choice prior to the influence of more extensive personal antenatal care experiences. Furthermore, limiting the sample to nulliparous women helped reduce potential bias from previous perinatal experiences.

### Measures

Data were collected through semi-structured interviews guided by an interview guide. A structured format was followed beginning with an introduction to welcome participants, explain the study objectives, and obtain informed consent, including explicit verbal consent for video recording. Prior to the interview, participants completed a brief questionnaire to collect demographic characteristics (age, relationship status, postal code, education level, and profession) and pregnancy-related information (gestational age, time to pregnancy, and miscarriage history). The core of the interview comprised fourteen open-ended questions, aiming to explore participants’ experiences, perceptions, and decision-making processes related to their selection of an ACP. Key topics included the timing and rationale behind ACP selection, experiences during consultations, expectations of antenatal care, and the perceived roles of different ACPs in managing low-risk pregnancies. Probing questions were used to elicit rich, detailed responses and clarify participant perspectives. Each interview concluded with a summary of key points discussed, an invitation for participant feedback, and an explanation of how the data would contribute to the research (Supplementary file S2).

To enhance construct validity, the interview guide was pretested with one pregnant woman who met the inclusion criteria. Received feedback on the clarity and structure of the questions was incorporated into the final version. In addition, the guide was reviewed and validated by two midwifery experts, with clinical experience in antenatal and perinatal care, as well as both the supervisors (MF and JV). During the initial interview, participants were also invited to comment on the interview process itself, specifically the structure, flow, and clarity of the questions to further ensure the appropriateness and comprehensibility of the guide.

The number of in-depth interviews was guided by the publication of Guest et al.^23^, who found that data saturation typically occurs after twelve interviews in a relatively homogeneous study population. Interviews continued until data saturation was reached, where no novel relevant knowledge could be identified.

### Procedure

Potential participants were invited to take part in the study through gatekeepers who were two independent midwives, one midwife working with an obstetrician, and one obstetrician. They received an information letter outlining the study’s aim and procedures. Low-risk pregnancies were identified based on the criteria outlined in the KCE (*Federaal Kenniscentrum voor de Gezondheidszorg*) report^24^, which provides a consensus framework for the referral and follow-up of low-risk pregnancies in primary care. This assessment was further informed by the ACP’s clinical expertise and aligned with the study’s inclusion and exclusion criteria. Participants were initially approached in person during their antenatal consultations, where they received information about the study and their potential role in it. Additionally, pregnant women interested in participating were able to contact the research team directly in response to a recruitment flyer.

The interviews were conducted by the first author (AC) with experience in qualitative research, between March and May 2024, and were visually recorded using Microsoft Teams^®^. Each interview lasted a maximum of 1.5 hours. During interviews, field notes were taken to document contextual factors, environmental disturbances, and to capture participants non-verbal cues from participants.

Member checking was conducted throughout the interviews through a process of ‘circling back’ to clarify participants’ interpretations, understanding, conclusions, and meanings using prompts such as: ‘Can you give an example of this?’, ‘What do you mean by that?’, ‘How did you feel in that situation?’. The interviewers intensively used facilitated communication techniques such as empathic paraphrasing, restatement, reflecting, and/or summarization to organize the received data and ensure accurate interpretation^25^.

### Ethics

Ethical approval was obtained from the Ethics Committee of UZ Brussel and Vrije Universiteit Brussel (VUB) (Approval number: B.U.N. 143.2023.0003.39; Date: 31 January 2024). Participants provided informed consent, including consent for audio and/or visual recording. Verbal consent was also obtained. Participants were informed of their right to withdraw at any time and assured of confidentiality. The interviewer disclosed her background in midwifery. The study followed the Declaration of Helsinki’s ethical guidelines throughout data collection and analysis^26^. The study was conducted in accordance with the ethical guidelines outlined in the Declaration of Helsinki ‘Good Clinical Practices’ and Belgian law.

### Data analysis

An inductive thematic analysis was conducted following the approach outlined by Braun and Clarke^27^. All recorded interviews were transcribed verbatim using Scribewave^®^, an online transcription service that converts audio and video recordings into text, thereby supporting accurate and efficient data processing for qualitative research. The interviewer verified the accuracy of the transcripts by reviewing them alongside field notes.

The first three interviews were independently coded line-by-line by three researchers (AC, MF, JV) leading to the development of initial codes. Based on this collaborative effort, an initial coding framework was constructed. Subsequently, AC continued the analysis by applying and refining the coding framework to the remaining interviews. Thereafter, codes were organized into potential themes, which were iteratively reviewed, refined, and defined and named.

To enhance internal reliability, regular briefing and debriefing sessions were held to discuss coding decisions, identify and refine candidate themes, and ensure consistency in interpretation. All researchers participated actively in reviewing and editing the written findings. Themes and sub-themes were further refined during a face-to-face consensus meeting involving all team members. To ensure analytical rigor, a systematic debriefing process was implemented, in which interpretations were critically examined, feedback was exchanged, and disconfirming evidence (i.e. deviant cases) was actively sought.

Anonymity was maintained throughout the transcription and analysis process. All interviews were conducted and analyzed in Dutch. For publication purposes, selected quotations were translated into English with close attention to preserving their original meaning and nuance. Data analysis was conducted in NVivo^®^ 14, a qualitative analysis software package.

## RESULTS

Thirteen nulliparous Flemish pregnant women participated in the in-depth interviews, with aged 25–34 years and gestational ages from 10 to 24 weeks. Four women were married, while the remaining nine were cohabiting with a partner. In terms of education, one participant had completed technical secondary education, nine held a Bachelor’s degree, and three had an Master’s degree. The time to conception ranged from almost immediate to three years. Additionally, five of the thirteen women had experienced at least one miscarriage. Table 1 provides a detailed overview of the participants’ demographic characteristics. To protect their privacy, the women were identified using codes (V1–V13).

**Table 1:** Sociodemographic characteristics of the study sample, Flanders, Belgium (N=13)

*Participant code*	*Age (years)*	*Education level*	*Profession*	*Marital status*	*Gestation week*	*Province*	*Duration to pregnancy*	*Possible miscarriages*	*Possible medical context of current pregnancy*
V1	30	Bachelor’s degree	Administrative employee	Married	24 weeks	Brussels Capital region	3 years	1 miscarriage after first blood test	Endometriosis (IVF)
V2	30	Bachelor’s degree	Kindergarten teacher	Living with partner	22 weeks	Flemish Brabant	Almost immediate	1 miscarriage at 8/9 weeks	-
V3	25	Bachelor’s degree	Lawyer	Married	12 weeks	Flemish Brabant	2 months	No	Current bleeding without further complications
V4	30	Master’s degree	Clerk	Married	20 weeks	Flemish Brabant	5 months	No	-
V5	34	Master’s degree	Clerk	Living with partner	13 weeks	Antwerp	8 months	No	-
V6	25	Bachelor’s degree	Personal coach/sports department employee	Living with partner	12 weeks	Flemish Brabant	Three inseminations (same-sex relationship)	1 miscarriage at 7.5 weeks	Pregnancy by insemination due to same-sex relationship
V7	31	Bachelor’s degree	Administrative employee	Living with partner	20 weeks	Antwerp	2 months	1 miscarriage at 6 weeks	Low platelets possibly due to autoimmune disease, but currently under control
V8	28	Bachelor’s degree	Talent acquisition partner	Living with partner	10 weeks	Antwerp	1 month	No	-
V9	28	Bachelor’s degree	Dietitian	Living with partner	20 weeks	Flemish Brabant	1 month	No	-
V10	29	Bachelor’s degree	Childcare assistant	Living with partner	22 weeks	Flemish Brabant	1.5 years	No	Asthma under medication but under control plus positive screening for preeclampsia in the first trimester
V11	28	Master’s degree	HR business partner	Married	19 weeks	East Flanders	3 months	No	Light bleeding at 5 weeks, everything under control
V12	27	TSO Secondary	Child supervisor	Living with partner	15 weeks	Antwerp	3 months	No	Hypothyroidism under medication but under control
V13	29	Bachelor’s degree	Speech therapist	Living with partner	21 weeks	East Flanders	4 months	1 miscarriage at 7 weeks	

Five major themes were identified from the data (Table 2). The themes provide insight into the interplay of personal beliefs, contextual constraints, and systemic structures influencing women’s reasons for choosing ACPs.

**Table 2:** Thematic summary of reasons for choosing an antenatal care provider, Flanders, Belgium (N=13)

*Theme*	*Description*
**Trust in professional expertise**	Women’s choices are shaped by perceived knowledge and professional competence of ACPs.
**Practical accessibility of care**	Logistical and time-related factors influence the choice, particularly ACP availability and convenience.
**Influence of social environment and informational gaps**	Decisions are shaped by social recommendations and limited knowledge about available care options.
**Need for relational continuity and emotional support**	A consistent care relationship and emotional comfort are prioritized during pregnancy.
**Structural and organizational constraints**	The extent of choice is often limited by how care is offered and communicated by ACPs.

ACP: antenatal care provider.

### Trust in professional expertise

A dominant theme was the perception of professional expertise, which influenced the decision-making process for many participants. Most women identified the obstetrician as the most qualified and reliable ACP. This perception was often rooted in the assumption that specialization equates to superior knowledge and safety. Several participants expressed concerns about delegating such an important responsibility to someone they perceived as less medically equipped:

*‘Health comes first. I wouldn’t risk my baby’s life by going to someone who knows less … I just want the one who knows the most, honestly.’* (V5)

Even when acknowledging that midwives have a medical degree and are trained in antenatal care, women unfamiliar with them before pregnancy had more trust in an obstetrician. Midwives were often described as playing a secondary or supportive role, rather than being capable of leading antenatal follow-up. In contrast, women who had experience with midwifery care or who received care in multidisciplinary settings tended to appreciate midwives’ competence and often viewed them as equally capable as obstetricians in managing normal pregnancies:

*‘I know the midwife has medical training too, but in my mind, it’s just not the same as a specialist.’* (V11)

*‘For me, there really is no difference between an obstetrician and a midwife. I honestly think they are both equally good.’* (V3)

GPs, on the other hand, were rarely considered as a primary ACP. Their broader generalist role was much appreciated, but not in the context of antenatal care:

*‘If I have something like the flu or a fever, I rely on my GP to prescribe the right medication and take my medical background into account. But when it comes to my pregnancy, I don’t really rely on my GP to get the right advice or input regarding my pregnancy.’* (V11)

Another dimension of this theme was the availability of technology and equipment. Several women preferred the obstetrician because they had direct access to ultrasound equipment and could offer immediate diagnostics, reducing the need for referrals:

*‘The obstetrician can do an ultrasound right away. At the GP, you have to be referred first, and that takes time.’* (V6)

This alignment of perceived expertise with technological access reinforced the medical authority of obstetricians. For many, technology became a symbol of safety and professionalism.

### Practical accessibility of care

Accessibility, both in terms of time and geography, emerged as a pragmatic but critical factor. Participants described how scheduling availability and convenience affected their choice. Care that required long travel times or excessive waiting was viewed as burdensome and was avoided when possible. In several cases, the GP was chosen for minor check-ups simply because it was faster and closer:

*‘At 16 weeks, the obstetrician said I could go to the hospital or to the GP for a urine and sugar check. My GP had already offered to do it, and I know her. It’s more practical and quicker than going to a big hospital where I don’t know anyone.’* (V5)

Speed and efficiency, especially in getting results or reassurance, were highly valued. Participants also described how medicalized institutions such as hospitals could sometimes feel overwhelming or impersonal, making them more inclined to choose smaller, more accessible practices.

Moreover, the timing of appointments was particularly important for women who experienced pregnancy-related anxiety or discomfort. Having to wait several weeks for reassurance could heighten stress levels, prompting them to choose an ACP who could see them sooner:

*‘A midwife might be a little more accessible than a gynecologist, right? You can reach out more easily and get quicker reassurance, whereas with a gynecologist, they’re often fully booked, and you might have to wait weeks for an appointment.’* (V8)

### Influence of social environment and informational gaps

Social influence was another key driver in women’s choices. Many women reported choosing their ACP based on recommendations from family members, friends, or peers. These endorsements were rarely questioned, particularly if the person recommending the ACP had positive outcomes during their own pregnancy:

*‘My sisters all went to this clinic and said it was great. They told me to go there, honestly I didn’t even know what a midwife really did back then.’* (V3)

This influence was particularly strong when women had no prior experience or pre-conceptional knowledge of the roles of different ACPs. In such cases, the default option was typically an obstetrician. Midwives were often overlooked simply because no one in their environment had spoken about them:

*‘Nobody around me talked about seeing a midwife, so it never crossed my mind.’* (V8)

In contrast, women who had friends or family who shared positive experiences with midwives became more open to considering them, even if their own ACPs had not presented midwifery care as an option. This highlights the central role of peer communication in shaping healthcare choices.

Women also reflected on the lack of structured information provided at the beginning of pregnancy. Very few recalled receiving a comprehensive overview of their options, which limited their ability to make informed choices:

*‘In my head it was just: I’m pregnant, so I go to the GP and make an appointment with the obstetrician. I think there could be more awareness [from ACPs] that there really are other [antenatal care] options, just like there are different options for birth itself.’* (V7)

Some women were surprised to learn about alternative antenatal care pathways, suggesting that information on available options is inconsistently communicated or fails to reach them:

*‘It’s not like there’s someone truly there for you who says, “Let’s go through all your options now”.’* (V5)

### Need for relational continuity and emotional support

The emotional dimension of antenatal care is as deeply important for women. Respondents expressed a strong preference for continuity of care – in their opinion, seeing the same ACP throughout pregnancy may help build trust, reduce stress, and foster a sense of security:

*‘Yes, I think that’s important. Just to have something to hold onto, that you feel good. That it’s familiar with those people, that it doesn’t add more stress.’* (V10)

Participants frequently contrasted the warm, personalized care provided by midwives with the more clinical or distant approach sometimes experienced with obstetricians. Midwives were seen as emotionally available, empathetic, and generous with their time:

*‘I have a softer feeling than with an obstetrician. They [midwives] are warmer, more emotional. A doctor is more analytical, and I think a midwife feels more personal, like a different kind of connection.’* (V7)

Hospital-based care was occasionally perceived as fragmented, rushed, or impersonal. Some women expressed discomfort with rotating staff or lack of continuity in larger institutions:

*‘You never know who you’re going to get at the hospital. It feels like you’re just a number.’* (V6)

Some participants also mentioned that emotional continuity with an ACP extended into the postpartum period, underscoring the long-term value of relational trust:

*‘Yes, I think it’s important, just to have some kind of support, so that you feel good, that it’s familiar. That it doesn’t cause more stress.’* (V10)

### Structural and organizational constraints

A final theme was the influence of healthcare system structures and how choices were presented, or not presented, to pregnant women. In most cases, women followed the pathway set out for them by their initial ACP, usually an obstetrician. Few women described being actively involved in a discussion about different types of ACPs or their roles:

*‘The obstetrician just told me “The next appointment will be with the midwife”* … *I went along with it.’* (V10)

This lack of transparency and shared decision-making meant that many women were unaware of how their care was being structured. While multidisciplinary collaboration was often appreciated, it was rarely initiated by the woman herself. Instead, it was presented as a predefined pathway with limited room for negotiation:

*‘Well, my obstetrician works with a midwife, and I don’t really have a say in that. The first appointments were with the midwife, and the first important ultrasound was with the obstetrician. So yes, they basically decide who you see when.’* (V2)

Some women attempted to seek out midwifery care early in pregnancy but were discouraged to wait until after 20 weeks, independently, by the midwife and the obstetrician. For example, one participant reported being told by a midwife to wait until 20 weeks, while another was advised by her obstetrician that it was not necessary to contact with a midwife until after the glucose test (between 24 and 28 weeks).

These system-level factors had a clear impact on whether women could exercise agency in choosing their ACP. The structure of the healthcare system, combined with a lack of proactive information-sharing by ACPs, often led to passive participation in the care process:

*‘But I think if I had another pregnancy, I’d involve a midwife sooner, because I didn’t know that was possible.’* (V9)

Moreover, women voiced a desire for standardized, accessible information about care trajectories. They emphasized the importance of understanding what to expect from each type of ACP, particularly in the early stages of pregnancy:

*‘*… *or that at the beginning of your pregnancy they would explain more clearly that you first go to the gynecologist and then to the midwife, that the midwife does this and that, but I think they sometimes assume that everyone knows that, while that’s not the case.’* (V10)

## DISCUSSION

The findings of this study reveal that the decision-making process of low-risk nulliparous women regarding antenatal care is multi-layered and shaped by a complex mix of perceptions, practicalities, social input, emotional needs, and structural realities. While some women expressed openness to alternative care models such as midwifery-led care, this openness was often constrained by a lack of awareness, system constraints, and reliance on habitual or externally recommended choices.

Our findings indicate that women’s perceptions of professional expertise and access to medical technology influenced their antenatal care choices. The preference for obstetricians appears to reflect common societal beliefs that associate specialist care with safety and professionalism, thereby reinforcing a medicalized approach to pregnancy. GPs were mainly perceived as managing only non-specialized, pregnancy-related concerns. This perception may contribute to how their role is understood within antenatal care and may influence the organization and accessibility of services. Similarly, most pregnant women in France considered pregnancy follow-up to fall within the competencies of obstetricians and midwives rather than GPs^16^. An Australian study also suggested that obstetricians are widely regarded by the public as the primary experts during the antenatal period^8^. Lambooij et al.^28^ found that Dutch pregnant women prioritized quality of care over provider type, supporting our finding that women’s preference for obstetricians is linked to perceptions of expertise and safety rather than profession alone. Comparable perceptions of expertise influencing provider preference were also observed in Canadian university students^17^. These findings highlight the significant role of perceived expertise, shaped by professional specialization and access to medical technology, in influencing women’s choices of ACPs.

In addition to perceptions of medical expertise, participants expressed varied views regarding the roles and professional competence of midwives. While some women who received care alternately from a midwife and an obstetrician viewed the midwife as equally skilled and professionally competent, while others perceived midwives as having more supportive role. Although midwives are the primary providers of care in many ANC settings^29^, they are often perceived as having secondary roles, highlighting a mismatch between perception and practice. Vermeulen et al.^4^ reported that younger Belgian women and those who have not yet given birth generally have less knowledge about the legal role of midwives. This may reflect gaps in women’s knowledge and a lack of clarity regarding the legal competencies of midwives, and their relative invisibility in antenatal care, highlighting the need for better communication and education about their professional roles.

Furthermore, logistical and time-related factors, such as appointment availability, location, and efficiency also influenced women’s choice of provider. The convenience of a nearby GP sometimes outweighed perceived expertise. Smaller practices were often preferred over hospital-based care due to shorter waiting times and a more personalized approach. These findings align with previous studies showing that trust, availability, proximity, and ease of follow-up influence women’s choice of an ACP^15,16^. While convenience and accessibility are important, they may challenge healthcare systems in balancing efficiency with continuity and comprehensive care. Therefore, these factors should be considered when designing antenatal services to support women’s informed choices.

The social environment and limited knowledge of available care options further contributed to women’s choice of ACPs. Family and peer recommendations mostly directed them toward obstetricians, while midwives remained less visible within their networks. Most participants perceived the GP and obstetrician as the standard option for antenatal care. Previous studies are in line with our findings, showing the role of the personal environment on the choice of an ACP^13,20^. Alzghoul et al.^10^ reported that some Canadian women selected a midwife as their ACP based on recommendations from friends or family members, and also emphasized the importance of choosing a female provider. Furthermore, Benahmed et al.^15^ noted that parents often lack information about which healthcare provider is responsible for monitoring a pregnancy. A US study also found that young women demonstrated considerable lack of knowledge or uncertainty regarding provider options for antenatal care^30^. These findings highlight how social environments can reinforce traditional pathways of care while limiting awareness of alternative care providers due to a lack of structured and accessible information, specifically at the beginning of pregnancy.

Relational continuity and emotional support also emerged as key factors influencing women’s decision-making regarding their choice of ACPs, with many highlighting the importance of personalized care. Midwives were commonly perceived as offering more individualized and relationship-based care compared with the more clinical approach of obstetricians^4,15^. A recent Cochrane review found that women in the United Kingdom, Canada, Australia, Ireland, and China who received midwife continuity of care models reported more positive antenatal care experiences compared with those in other models^31^. Similarly, Talrich et al.^32^ reported that Belgian women value Group Antenatal Care (GANC), an alternative model that combines interactive learning, continuous support, and health assessment conducted by an ACP (e.g. an obstetrician, midwife, or GP). Although GANC is still relatively new in most Western countries, the integration of GANC or midwife continuity of care models into traditional care pathways may better meet pregnant women’s needs for continuity and support, while complementing existing services. This integration could influence women’s choice of care provider by aligning service delivery with their expectations for a more personalized and supportive approach. It may also inform policy decisions aimed at optimizing care pathways to more accurately reflect women’s needs and preferences.

Women in this study reported that structural and organizational constraints within the healthcare system influenced their choice of ACPs and expectations for accessible and standardized care options. Although participants expressed satisfaction with receiving care from multiple ACPs, the lack of transparency and poor communication among providers emerged as barriers to shared decision-making regarding care options. Similarly, previous research has highlighted how organizational constraints, such as limited access to resources, inadequate training, and resistance to new guidelines, can shape women’s choices of ACPs. System-level challenges, including insufficient management, scarcity of facilities, and the lack of continuous, accessible care models, may also influence these choices^11,33,34^. Lambooij et al.^28^ further showed that in the Netherlands, organizational models such as bundled payment and structured care can limit women’s choice of provider. From these findings, it can also be concluded that women are open to prenatal care pathways involving multiple providers. However, receiving care from multiple ACPs alone is not sufficient to ensure effective multidisciplinary collaboration unless supported by clear structures and robust communication systems.

### Strengths and limitations

To our knowledge, this is a novel study to explore why Flemish low-risk nulliparous pregnant women choose a specific ACP for antenatal care in Belgium. The study’s qualitative descriptive design enabled an in-depth exploration of these women’s experiences and perceptions. This approach facilitated the development of rich, detailed narratives that uncovered nuanced insights into their decision-making process, understandings that may not captured through quantitative studies. While contextual factors are specific to Flanders, the identified themes are relevant across healthcare systems and contribute to international discussions on women’s autonomy and the role of ACPs in antenatal care.

However, this study has several limitations. Although recruitment primarily occurred through gatekeepers, some participants were recruited through personal networks and snowball sampling, which may have introduced selection bias. Furthermore, the findings are interpretive in nature and do not allow causal relationships between factors influencing antenatal care provider choice to be established. Finally, participants were interviewed at a single point during pregnancy, and their views and preferences may have evolved over time.

## CONCLUSIONS

Our findings indicate that Flemish nulliparous pregnant women’s choice of an antenatal care provider is shaped by multiple interacting factors, including perceived professional competence, logistical and time-related factors, social recommendations, limited awareness of available care options, relational continuity, emotional support, and organizational structures.

Obstetricians were commonly viewed as the primary and most reliable care providers. However, awareness of available ACP options, such as midwives and general practitioners, was limited, and women’s choices were often influenced by their social environment. Midwives were mainly associated with information and psychological support, while GPs were seen as providing general, less specialized care. Overall, women valued continuity, accessibility, multidisciplinary collaboration, and effective communication between providers.

Further research is needed to explore healthcare professionals’ perspectives on low-risk antenatal care pathways and to examine how different models of care influence women’s decision-making and experiences.

## Supplementary Material



## Data Availability

The data supporting this research are available from the authors on reasonable request.
