# Supplementary material for: The reasons why Flemish low-risk nulliparous pregnant women choose a specific antenatal care provider: A descriptive qualitative study
Source: Eur J Midwifery. 2026 Oct 15;10:10.18332/ejm/224241. doi: 10.18332/ejm/224241 (PMC13670242; doi:10.18332/ejm/224241)
Supplement: Supplementary file 1 [file EJM-10-48-s1.pdf]

EUROPEAN JOURNAL
OF MIDWIFERY

Supplementary file

© 2026 Cloostermans A.
et al.

DOI:
10.18332/ejm/224241

The content has been provided by the author(s) and has not been reviewed, verified, or endorsed by European Publishing. It may not have undergone peer review. The views, opinions, and recommendations expressed are solely those of the author(s) and do not necessarily reflect the position of European Publishing. European Publishing accepts no responsibility or liability for any consequences arising from the use of, or reliance on, this content.

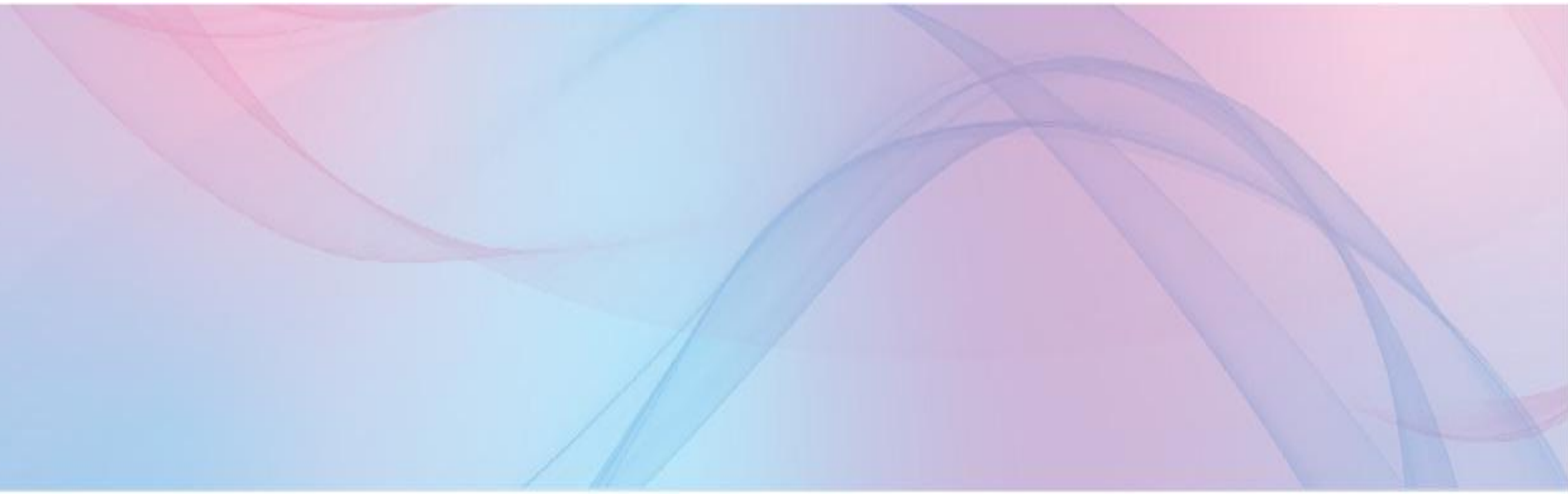

Supplementary File S1: Standards for Reporting Qualitative Research (SRQR)*

Title and abstract

**Title** - Concise description of the nature and topic of the study Identifying the study as qualitative or indicating the approach (e.g., ethnography, grounded theory) or data collection methods (e.g., interview, focus group) is recommended	1
**Abstract** - Summary of key elements of the study using the abstract format of the intended publication; typically includes background, purpose, methods, results, and conclusions	1

Introduction

**Problem formulation** - Description and significance of the problem/phenomenon studied; review of relevant theory and empirical work; problem statement	1,2
**Purpose or research questio**n - Purpose of the study and specific objectives or questions	2

Methods

**Qualitative approach and research paradigm** - Qualitative approach (e.g., ethnography, grounded theory, case study, phenomenology, narrative research) and guiding theory if appropriate; identifying the research paradigm (e.g., postpositivist, constructivist/ interpretivist) is also recommended; rationale**	2
**Researcher characteristics and reflexivity** - Researchers' characteristics that may influence the research, including personal attributes, qualifications/experience, relationship with participants, assumptions, and/or presuppositions; potential or actual interaction between researchers' characteristics and the research questions, approach, methods, results, and/or transferability	2,3
**Context** - Setting/site and salient contextual factors; rationale**	2,3
**Sampling strategy** - How and why research participants, documents, or events were selected; criteria for deciding when no further sampling was necessary (e.g., sampling saturation); rationale**	3
**Ethical issues pertaining to human subjects** - Documentation of approval by an appropriate ethics review board and participant consent, or explanation for lack thereof; other confidentiality and data security issues	3
**Data collection methods** - Types of data collected; details of data collection procedures including (as appropriate) start and stop dates of data collection and analysis, iterative process, triangulation of sources/methods, and modification of procedures in response to evolving study findings; rationale**	2,3
**Data collection instruments and technologies** - Description of instruments (e.g., interview guides, questionnaires) and devices (e.g., audio recorders) used for data collection; if/how the instrument(s) changed over the course of the study	2,3

**Units of study** - Number and relevant characteristics of participants, documents, or events included in the study; level of participation (could be reported in results)	2,3
**Data processing** - Methods for processing data prior to and during analysis, including transcription, data entry, data management and security, verification of data integrity, data coding, and anonymization/de-identification of excerpts	3
**Data analysis** - Process by which inferences, themes, etc., were identified and developed, including the researchers involved in data analysis; usually references a specific paradigm or approach; rationale**	3
**Techniques to enhance trustworthiness** - Techniques to enhance trustworthiness and credibility of data analysis (e.g., member checking, audit trail, triangulation); rationale**	3

Results/findings

**Synthesis and interpretation** - Main findings (e.g., interpretations, inferences, and themes); might include development of a theory or model, or integration with prior research or theory	3-6
**Links to empirical data** - Evidence (e.g., quotes, field notes, text excerpts, photographs) to substantiate analytic findings	3-6

Discussion

**Integration with prior work, implications, transferability, and contribution(s) to the field -** Short summary of main findings; explanation of how findings and conclusions connect to, support, elaborate on, or challenge conclusions of earlier scholarship; discussion of scope of application/generalizability; identification of unique contribution(s) to scholarship in a discipline or field	6-8
**Limitations** - Trustworthiness and limitations of findings	8

Other

**Conflicts of interest** - Potential sources of influence or perceived influence on study conduct and conclusions; how these were managed	10
**Funding** - Sources of funding and other support; role of funders in data collection, interpretation, and reporting	10

*The authors created the SRQR by searching the literature to identify guidelines, reporting standards, and critical appraisal criteria for qualitative research; reviewing the reference lists of retrieved sources; and contacting experts to gain feedback. The SRQR aims to improve the transparency of all aspects of qualitative research by providing clear standards for reporting qualitative research.

**The rationale should briefly discuss the justification for choosing that theory, approach, method, or technique rather than other options available, the assumptions and limitations implicit in those choices, and how those choices influence study conclusions and transferability. As appropriate, the rationale for several items might be discussed together.

Supplementary File S1: Standards for Reporting Qualitative Research (SRQR)*

Reference:

O'Brien, B. C., Harris, I. B., Beckman, T. J., Reed, D. A., & Cook, D. A. (2014). Standards for reporting qualitative research: a synthesis of recommendations. Academic medicine : journal of the Association of American Medical Colleges, 89(9), 1245–1251. https://doi.org/10.1097/ACM.0000000000000388

Supplementary File S2: Interview Guide for In-Depth Interviews

Introduction

- Welcome and thank the participant for their participation.
- Introduce researcher, the research topic, and the aim of the interview.
- Explain the interview process and duration (approximately 1.5 hours).
- Assure anonymity and explain participant rights.
- Ask if the participant has any questions or concerns.
- Request consent for video recording.
- Start the recording.
- Obtain explicit verbal consent for data collection and processing, including consent for video recording, by reviewing the informed consent form.
- Begin with the brief oral questionnaire.

Participant Characteristics Questionnaire (To be asked orally before the interview)

- What is your age?
- What is your family situation? (Single, cohabiting, married, etc.)
- What is your postal code?
- What is your educational level? (Primary education, vocational secondary, technical secondary, general secondary, associate degree, professional bachelor's, academic bachelor's, master's, PhD, etc.)
- What is your profession?
- How many weeks pregnant are you?
- How long did it take you to become pregnant?
    - Have you had one or more miscarriages?
    - If yes, how many and at what stage of pregnancy?

Interview Opening Questions:

- How are you feeling in your pregnancy right now?
    - To what extent does this align with your expectations before becoming pregnant?

Core Questions: Facilitators and Barriers in Choosing a Healthcare Provider

- Which type(s) of healthcare provider have you consulted for your prenatal care so far?

    - At what stage of your pregnancy did you consult [healthcare provider(s) mentioned]?
    - How did you generally feel about [healthcare provider(s) mentioned]?
    - Was there a specific reason why you chose [healthcare provider(s) mentioned] for your prenatal care?
- Can you describe how your first consultation with your healthcare provider went?
- Which healthcare provider(s) do you plan to visit for the remainder of your pregnancy?
    - At what stage do you plan to visit [healthcare provider(s) mentioned]?
    - Is there a specific reason why you have chosen to continue your prenatal care with [healthcare provider(s) mentioned]?
- What are your thoughts on the number of consultations and their distribution throughout pregnancy?
    - How do you feel about receiving prenatal care from multiple healthcare providers?
    - To what extent would a midwife add value to your prenatal care?
        - [If applicable] At what stage of pregnancy?
    - To what extent does the cost of care influence your choice of healthcare provider for prenatal care?
- [If applicable] Why did you choose a combination of [type of healthcare provider] and [type of healthcare provider] for your prenatal care?
    - [If applicable] How was the communication between healthcare providers?
        - What, in your opinion, facilitated communication between providers?
        - What, in your opinion, hindered communication between providers?
        - What could help improve communication?

Role of Healthcare Providers in Prenatal Care

- In your view, what does quality prenatal care look like?
- Can you describe potential advantages of receiving prenatal care from a gynecologist for a low-risk pregnancy?
    - Can you describe potential disadvantages?
- Can you describe potential advantages of receiving prenatal care from a midwife for a low-risk pregnancy?

  - Can you describe potential disadvantages?
- Can you name three words or phrases that come to mind when you think of a gynecologist?
  - Can you name three words or phrases that come to mind when you think of a midwife?
- In your opinion, what role should a gynecologist play in a low-risk pregnancy?
  - What role should a midwife play?
  - What role should a general practitioner play?
- What expectations do you have from your chosen healthcare provider(s) throughout your pregnancy?
  - To what extent have your expectations for prenatal care been met so far?
- When thinking about your upcoming birth and the postpartum period:
  - How important is it to you to be followed by the same healthcare provider(s) throughout your pregnancy, birth, and postpartum period?
- Would you choose the same healthcare provider(s) again for a future pregnancy?
  - [If applicable] Why?
  - [If applicable] Why not?

Conclusion / Completion

- Summarize the key points of the interview.
- Ask if there is anything else that was missed.
- Ask for feedback on the interview.
- Ask what the most important takeaway from the discussion was for the participant.
- Thank the participant for their time and contribution.
- Provide the researcher's contact details in case of any follow-up questions.
- Explain how the interview results will be published.

Examples of Follow-Up Questions:

- Can you give an example of this?
- What do you mean by that?
- How did you feel in that situation?
